# Antimicrobial resistance among clinically relevant bacterial isolates in Accra: a retrospective study

**DOI:** 10.1186/s13104-018-3377-7

**Published:** 2018-04-25

**Authors:** Jibril Mohammed, Yaovi Mahuton Gildas Hounmanou, Line Elnif Thomsen

**Affiliations:** 1 Department of Microbiology, Parasitology and Biotechnology, College of Veterinary Medicine and Biomedical Sciences, Sokoine University of Agriculture, Morogoro, Tanzania; 2 Department of Veterinary and Animal Sciences, Faculty of Health and Medical Sciences, University of Copenhagen, 1870 Frederiksberg, Denmark

**Keywords:** Antimicrobial resistance, Clinical bacteria, Ghana

## Abstract

**Objective:**

The aim of this study was to determine the antimicrobial resistance pattern of bacterial isolates from different specimens at various hospitals and private diagnostic service laboratories in Ghana.

**Results:**

A retrospective data of culture and sensitivity test results from 2016 were extracted from the microbiology record book of six laboratories in Accra, Ghana. The data included type of clinical specimen, sex of patient, name of bacterial isolate and antibiotic resistance profile. A total of 16.6% (n = 10,237) resistant isolates were obtained, however, the proportions of resistant isolates varied significantly between laboratories. High resistance towards tetracycline, ampicillin, cotrimoxazole and cephalosporins, but low towards amoxiclav and aminoglycosides, was observed. This study identified *E. coli* and *Staphylococcus* species as the major resistant bacteria from clinical specimen in Accra and the highest prevalence of the isolates was found in urine specimens in all six laboratories (69.1%, n = 204; 52.6%, n = 36; 52.3%, n = 350; 37.9%, n = 298; 53%, n = 219; 62.1%, n = 594) and in female patients (81.4, 50 and 69.5%). Regular surveillance and local susceptibility pattern analysis is extremely important in selecting the most appropriate and effective antibiotic for the treatment of bacterial infections.

**Electronic supplementary material:**

The online version of this article (10.1186/s13104-018-3377-7) contains supplementary material, which is available to authorized users.

## Introduction

One of modern medicine’s greatest achievements has been the production of antimicrobials against disease-causing microbes, but after more than 70 years of widespread use, these therapeutic agents have gradually lost their potency [1]. Antimicrobial resistance (AMR) is now a serious global health concern causing problems in the treatment and prevention of infections. Nevertheless, these microorganisms especially bacteria, causes some of the most common infections in different settings; in the community, in hospitals or transmitted through the food chain [2]. Antibiotics are among the most commonly prescribed drugs in hospitals and in developed countries about 30% of the hospitalized patients are treated with these drugs [3]. Antibiotic use in Africa is progressively on the rise and the availability of un-prescribed antibiotics is part of the problem. A study combining data from various African countries revealed that approximately 90.1% of cases of acute illness sought care outside the home and 36.2% took an antibiotic medication and over 30% of the individuals acquired antibiotics without prescription [4]. Several studies from various African countries have furthermore shown high levels of antibiotic resistant bacterial pathogens [5–9]. In spite of the ongoing research on antimicrobial resistance, there are still no indications that the situation is abating. In order to formulate an efficient AMR control plan, it is crucial to have a clear view on the current situation so as to determine when, how and where to initiate control measures. The present study assessed a 12 months trend of antimicrobial resistance in clinically relevant bacterial isolates in Accra, Ghana.

## Main text

### Methods

#### Study design

This study was a retrospective analysis of miscellaneous clinical samples that were tested for bacterial presence and subsequent susceptibility testing dated from January to December 2016, from six locations comprising three hospitals and three private diagnostic service laboratories. The study locations include Trust Specialist Hospital (TH), Holy Trinity Medical Center (HTMC), LA General Hospital (LAGH), Patholab Solutions (Ghana) Limited (PSGL), G2 Medical Laboratory and Mediplast Diagnostic Center (G2ML). All locations are situated in the city of Accra, Ghana and the data were sampled from January to May 2017.

#### Data extraction

The clinical information extracted from the six laboratories included type of sample analyzed, name of pathogens isolated and the names of antibiotics used for susceptibility testing and the susceptibility results as recorded in the laboratory report. All information were confirmed by each laboratory technician and reconfirmed by the chief biomedical scientist.

#### Laboratory analysis, clinical samples and collection of bacterial isolates

Several types of clinical samples were cultured, including urine, blood, sputum, urethral smear, pus and seminal fluid, and swabs from various body sites (vagina, ear, throat, umbilical cord, eye and wound). All the laboratories sampled for the current study employed similar standard microbiological culturing techniques.

#### Bacterial identification

All the laboratories performed similar microscopic identification as previously described [10]. For Gram-positive cocci, tube coagulase and catalase were done for species differentiation. Further biochemical tests included lactose fermentation, indole, citrate utilization, urease and Triple Sugar Iron reaction to ascertain the biochemical characteristics of the Gram-negative isolates. Two of the laboratories (Trust Hospital and Mediplast Diagnostic Center) also deployed the BBL Crystal Panel Viewer (Becton–Dickinson Maryland, USA) for further identification of isolates.

#### Antimicrobial susceptibility

Antimicrobial susceptibility test were performed by all 6 laboratories using the Kirby-Bauer disk diffusion method [11] and interpreted using the Clinical Laboratory Standard Institute guidelines [12]. Gram-positive and Gram-negative antibiotic disks were selected for Gram-positive and Gram-negative bacterial isolates, respectively. Each laboratory used disks manufactured by Abtek Biologicals, UK and Biomark Laboratories, India. The disks and their concentrations in micrograms included: ampicillin (AMP: 10), cotrimoxazole (COT: 25), amoxiclav (AMC: 20/10), nalidixic acid (NAL: 30), tetracycline (TET: 30), ceftriaxone (CTR: 30), ceftazidime (CAZ: 30), nitrofurantoin (NIT: 300), gentamicin (GEN: 10), ciprofloxacin (CIP: 30), levofloxacin (LEV: 5), norfloxacin (NOR: 30), chloramphenicol (CHL: 10), cefuroxime (CRX: 30), amikacin (AMK: 30), piperacillin (PIP: 100), nitrofurantoin (NTD: 200), cefotaxime (CTX: 30), penicillin (P: 1.5), flucloxacillin (FLX: 1), erythromycin (ERY: 5), amoxicillin (AMX: 30), ofloxacin (OF: 5), cloxacillin (CLX: 5), perfloxacin (PEF: 10), augmentin (AUG: 30), vancomycin (VAN: 30), meropenem (MEM: 10), roxithromycin (RO: 15), sparfloxacin (SPX: 5), lincomycin (LM: 15), azithromycin (AZM: 15), pipemidic acid (PPA: 20).

#### Data analysis

Collected data was entered into Microsoft Excel and loaded into statistical package for social sciences (SPSS, version 20) for analysis. Proportions of predominant isolates and antibiotic resistance profiles were compared using Chi square test. The Pearson correlation test was used to assess associations among locations and isolates in relation to the resistance profile of the isolates at a critical probability *P *< 0.05.

### Results

Records on resistant bacterial isolates and the antibiotic resistant profile cultured from January to December 2016 in six microbiology laboratories were extracted. These laboratories include three hospitals namely Trust Hospital (TH), LA General Hospital (LAGH) and Holy Trinity Medical Center (HTMC) and three private diagnostic service laboratories, Mediplast Diagnostic Center (MDC), Patholab Solutions Ghana Limited (PSGL) and G2 Medical Laboratory (G2ML).

In the study period, a total of 10,237 samples were cultured and 1701 (16.62%) resistant isolates were obtained. However, proportions of antibiotic resistant bacteria differed significantly from one laboratory to the other with PSGL samples generating the highest number of resistant isolates (33.08%, P < 0.05), whereas isolates recovered at G2ML showed the lowest level of resistance (5.51%, P < 0.05) (Table 1).

**Table 1:** Comparison of antibiotic resistance between laboratories using Chi square test

Laboratories	Number of isolates	Resistant isolates (%)
TH	1515	204 (13.47^d^)
MDC	243	36 (14.81^d^)
PSGL	1058	350 (33.08^a^)
LAGH	1302	298 (22.89^c^)
G2ML	3978	219 (5.51^e^)
HTMC	2141	594 (27.74^b^)

Proportions followed by different letters in a column means differences in proportion of resistant isolates at α < 0.05%

Out of the six labs, nineteen different specimens were taken and statistical analysis revealed from all laboratories that urine samples are significantly more contaminated with antibiotic resistant bacteria than all other sample types (P < 0.05, Additional file 1).

Samples were taken from female and male patients in all the six study areas but only three provided data on sex groups. A two by two comparison revealed for TH and LAGH that female gender is significantly associated with infection by antibiotic resistant bacteria compared to males (P < 0.05, Table 2). MDC had equal proportions (50%) of resistant bacteria from male and female patients (Table 2).

**Table 2:** Repartition of resistant bacteria by sex of patients

Laboratories	Resistant isolates		P-value
	Females (%)	Males (%)	
TH	166 (81.37)	38 (18.63)	0.000001
MDC	18 (50)	18 (50)	0.8136
LAGH	207 (69.46)	91 (30.54)	0.000001

*TH* Trust Hospital, *MDC* Mediplast Diagnostic Center, *LAGH* LA General Hospital

The predominant resistant bacterial species varied from one lab to the other, but *E. coli* and *Staphylococcus* were the main isolates from all the laboratories. From TH and G2ML, *E. coli* were the most recovered isolates (47.5%, n = 204 and 38.4%, n = 219, respectively). However, *S. aureus* prevailed in isolates recovered at MDC, PSGL and LAGH (22.2%, n = 36; 50.9%, n = 350 and 29.5%, n = 298, respectively). Undistinguished resistant Staphylococcus species were the most common isolates from HTMC (44.8%, n = 594) (Table 3).

**Table 3:** Proportion of resistant bacterial species isolated from clinical samples from each laboratory

Resistant isolates	TH	MDC	PSGL	LAGH	G2ML	HTMC
	Number (%)	Number (%)	Number (%)	Number (%)	Number (%)	Number (%)
*Providencia* spp.	NR	NR	NR	NR	NR	53 (8.9)
*M. morganii*	NR	NR	NR	NR	NR	3 (0.5)
*Citrobacter* spp.	NR	1 (2.8)	1 (0.3)	17 (5.7)	23 (10.5)	NR
*S. aureus*	21 (10.3)	8 (22.2)	178 (50.9)	88 (29.5)	28 (12.8)	NR
*Pseudomonas* spp.	16 (7.8)	3 (8.3)	13 (3.7)	29 (9.7)	25 (11.4)	7 (1.2)
*S. epidermidis*	3 (1.5)	4 (11.1)	1 (0.3)	1 (0.3)	6 (2.7)	NR
*K. pneumoniae*	NR	1 (2.8)	NR	1 (0.3)	NR	NR
*P. aeruginosa*	NR	1 (2.8)	3 (0.9)	18 (6.0)	14 (6.4)	12 (2.0)
*Enterobacter* spp.	6 (2.9)	5 (13.9)	NR	12 (4.0)	3 (1.4)	23 (3.9)
*N. gonorrhoea*	4 (2.0)	2 (5.6)	NR	NR	12 (5.5)	NR
*E. coli*	97 (47.5)	7 (19.4)	120 (34.3)	6.8 (22.8)	84 (38.4)	86 (14.5)
*Y. pestis*	NR	1 (2.8)	NR	NR	NR	NR
*S. pyogenes*	NR	NR	NR	NR	NR	2 (0.3)
*Proteus* spp.	5 (2.5)	2 (5.6)	7 (2.0)	17 (5.7)	NR	4 (0.7)
*Klebsiella* spp.	25 (12.3)	1 (2.8)	4 (1.1)	20 (6.7)	1 (0.5)	12 (2.0)
*P. vulgaris*	NR	NR	8 (2.3)	NR	1 (0.5)	NR
*S. saprophyticus*	NR	NR	7 (2.0)	6 (2.0)	NR	NR
*E. faecalis*	27 (13.2)	NR	7 (2.0)	NR	NR	NR
*P. mirabilis*	NR	NR	1 (0.3)	8 (2.7)	2 (0.9)	NR
*Enterococcus* spp.	NR	NR	NR	6 (2.0)	14 (6.4)	31 (5.2)
*Acinetobacter* spp.	NR	NR	NR	1 (0.3)	1 (0.5)	NR
*Bacillus* spp.	NR	NR	NR	3 (1.0)	NR	1 (0.2)
*Streptococcus* spp.	NR	NR	NR	2 (0.7)	5 (2.3)	NR
*Salmonella* spp.	NR	NR	NR	1 (0.3)	NR	NR
*Staphylococcus* spp.	NR	NR	NR	NR	NR	266 (44.8)
*C. freundii*	NR	NR	NR	NR	NR	94 (15.8)

*NR* not reported, *TH* Trust Hospital, *MDC* Mediplast Diagnostic Center, *PSGL* Patholab Solutions (Ghana) Limited, *LAGH* LA General Hospital, *G2ML* G2 Medical Laboratory Service, *HTMC* Holy Trinity Medical Centre

Records of the bacterial species and their resistance pattern can be found in Additional file 2. Here we summarized the antibiotic resistance profile of the three predominant resistant isolates from each laboratory. At TH, *E. coli*, *Enterococcus faecalis* and *Klebsiella* spp were resistant to AMP, COT, AMC, NAL, TET, CRO, GEN, LEV, NOR, CXM, NTD and CTZ at varying proportions. At MDC, *S. aureus, E. coli and Enterobacter* spp displayed resistance of varying degrees to AMP, COT, TET, CIP, NOR, CHL and CXM. Data from PSGL show that *S. aureus, E. coli and Pseudomonas* spp demonstrated resistance to CEF, PIP, CHL, CTX, AMK, GEN, NAL, CFT, AUG, CIP, TET, NIT, VAN, CTR, MEM, COT, ERY AMP and CRX. From LAGH, the predominant isolates (*S. aureus, E. coli and Pseudomonas* spp) were resistant to COT, CIP, GEN, AMP, MEM, TET, CRX, AMK, LEV, CTR and CTX. At G2ML *S. aureus, E. coli and Citrobacter* spp showed resistance against GEN, CIP, AMP, AUG, COT, CRX, TET, CFT, PIP, NAL, CTX, CTR and CHL. The three predominant isolates (*E. coli*, *Staphylococcus* spp and *Citrobacter freundi*) from HTMC were resistant to CPZ, CIP, CTR, PIP, CTX, TET, LEV, GEN, NX, AMK, NA, AMP, OF, CHL, CXM, COT, NIT, AMX, ERY, LM, RO and AZM with different proportions (Additional file 2).

### Discussion

Antibiotic resistant bacterial infections have become a threat, in particular in developing countries, but to obtain an effective treatment plan, it is vital to have an overview of the current resistance level. In this study, data on culture and sensitivity results from six microbiology laboratories were extracted. Within the entire period, each of the six laboratories reported varying numbers of antibiotic resistant bacteria strains ranging from 36 to 594 resistant isolates. Statistical analysis revealed that samples from PSGL generated the highest proportions of resistant isolates (33.08%). There is therefore a possibility that patients living around or attending PSGL are likely to be more infected with antibiotic resistant bacteria, as it is situated in a densely populated area of residence, compared to patients who attended the other laboratories. Isolates recovered at G2ML, located in an area which primarily covers a business district, demonstrated the lowest level of antibiotic resistance as compared to the rest (5.51%). Among the six labs, 19 different types of clinical specimen were processed, but the highest level of antibiotic resistant bacteria was found in the urine samples in all laboratories. The proportions of resistant isolates ranged from 37.9% and up to 69.1%. High level resistance (66.7%) in urine has also been shown at the Korle-Bu Teaching Hospital in Ghana [13]. In Sierra Leone 85.7% of multidrug resistant isolates were identified in urine specimen [14]. Furthermore, the most prevailing resistant bacteria isolated from the various specimens in all the laboratories were *E. coli* and Staphylococcus. *E. coli* is the primary etiologic agent causing urinary tract infection, accounting for 90% of the cases [15], however, in this study we did not acquire data on disease state of patients. But our data showed that female patients from TH (81.37%) and LAGH (69.46%) had the highest level of resistant bacteria compared to males. That significantly more resistant bacteria were found in female urine samples correlates with a higher susceptibility of infection in females than males, due to the physiological and anatomical differences. Resistant *Staphylococcus aureus* recovered from various specimens were found in the range 10.3–50.9%. The undistinguished *Staphylococcus* spp. (44.8%) from HTMC laboratory may be due to lack of special media or other requirements for accurate and definitive identification. High level antibiotic resistant *S. aureus* has previous been shown in other studies from Ghana and Ethiopia [5, 16, 17]. Often, high levels of *S. aureus* are isolated from different sites of infection, probably due to the fact that this bacterium is part of the normal flora on skin and gut, but can infect breaches on the skin. Moreover, *S. aureus* is often found at the hospital settings, increasing the risk of infections. Due to the known risk of bacterial spread at hospitals, and lack of typing data, we cannot rule out that some of the resistant bacteria isolated from different specimen may in fact be the same clone.

Gram-negative bacteria accounted for 65.4% of the resistant bacteria, whereas Gram-positive accounted for 34.6%. Overall, *S. aureus*, *E. coli*, *Citrobacter* spp., *Pseudomonas* spp. and *Enterobacter* spp. from all the laboratories, showed high resistance against tetracycline, ampicillin and cotrimoxazole. Similar report of high resistance to these antibiotics in Ghana has previously been reported [5, 18]. They are also regarded as the most widely used antibiotics in developing countries, as they are considered inexpensive and generally have broad-spectrum activity [19]. We also observed high resistance of *Klebsiella* spp. to ceftriaxone and cefuroxime, which has also been observed in a recent study from Rwanda [20]. But the resistance to amoxiclav, amikacin and chloramphenicol was low across most of the laboratories except at G2ML where resistance towards amoxiclav (augmentin) was mostly high.

### Conclusion

Our analysis of the culture and sensitivity test results of the various species of bacteria isolated from different clinical specimens at various microbiology laboratories revealed that high levels of resistant bacteria were recovered from urine specimens. Furthermore, resistant *E. coli* and *Staphylococcus* species were the most prevalent isolates recovered from all the specimens. Specimen from female patients presented the highest prevalence of resistant bacteria isolates as compared to male patients. We observed high resistance to tetracycline, ampicillin, cotrimoxazole and the cephalosporins, but low resistance to amoxiclav and the aminoglycosides. The variations in resistance pattern of the bacterial isolates across the laboratories suggests that regular surveillance and local susceptibility pattern is extremely important in selecting the most appropriate and effective antibiotic for the treatment of bacterial infections.

## Limitations of the study

Our study have some limitations due to lack of information on the disease status of the patients, and it is possible that different specimens were collected from the same patient, resulting in isolation of the same bacterial species.

## Additional files

**Additional file 1.** Antibiotic resistant bacteria isolated from different sample types at the six laboratories. Total number and percentage of resistant bacterial isolates from each type of specimen isolated at TH (Trust Hospital); MDC (Mediplast Diagnostic Center); PSGL (Patholab Solutions Ghana Limited); LAGH (LA General Hospital); G2ML (G2 Medical Laboratory); HTMC (Holy Trinity Medical Center).

**Additional file 2.** List of isolates and proportion (%) of resistance against each tested antibiotic. Data shown for each laboratory. Total list of all bacteria isolated at the six laboratories and the percentage of resistant bacteria against all tested antibiotics. TH (Trust Hospital); MDC (Mediplast Diagnostic Center); PSGL (Patholab Solutions Ghana Limited); LAGH (LA General Hospital); G2ML (G2 Medical Laboratory); HTMC (Holy Trinity Medical Center).

## Abbreviations

TH: Trust Hospital
MDC: Mediplast Diagnostic Center
PSGL: Patholab Solutions Ghana Limited
LAGH: LA General Hospital
G2ML: G2 Medical Laboratory
HTMC: Holy Trinity Medical Center
AMR: antimicrobial resistance
SPSS: statistical package for social sciences
AMP: ampicillin
COT: cotrimoxazole
AMC: amoxiclav
NAL: nalidixic acid
TET: tetracycline
CTR: ceftriaxone
CAZ: ceftazidime
NIT: nitrofurantoin
GEN: gentamicin
CIP: ciprofloxacin
LEV: levofloxacin
NOR: norfloxacin
CHL: chloramphenicol
CRX: cefuroxime
AMK: amikacin
PIP: piperacillin
NTD: nitrofurandantoin
CTX: cefotaxime
P: penicillin
FLX: flucloxacillin
ERY: erythromycin
AMX: amoxicillin
OF: ofloxacin
CLX: cloxacillin
PEF: perfloxacin
AUG: augmentin
VAN: vancomycin
MEM: meropenem
RO: roxithromycin
SPX: sparfloxacin
LM: lincomycin
AZM: azithromycin
PPA: pipemidic acid

## References

[1] Kimanga AN (2012). A situational analysis of antimicrobial drug resistance in Africa: are we losing the battle?. Ethiop J Health Sci..

[2] Antimicrobial Research Council. Antimicrobial resistance. 2014. http://www.mrc.ac.uk/documents/pdf/antimicrobial-resistance-timeline-report/. Accessed 4 Aug 2017.

[3] Shankar RP, Partha P, Shenoy NK, Easow JH, Brahmadathan KN (2003). Prescribing patterns of antibiotics and sensitivity patterns of common microorganisms in the Internal Medicine ward of a teaching hospital in Western Nepal: a prospective study. Ann Clin Microbiol Antimicrob..

[4] Vialle-Valentin CE, Lecates RF, Zhang F, Desta AT, Ross-Degnan D (2012). Predictors of antibiotic use in African communities: evidence from medicines household surveys in five countries. Trop Med Int Health..

[5] Newman MJ, Frimpong E, Donkor ES, Opintan J (2011). Resistance to antimicrobial drugs in Ghana. Infect Drug Resist..

[6] Dada-Adegbola H, Muili K (2010). Antibiotic susceptibility pattern of urinary tract pathogens in Ibadan, Nigeria. Afr J Med Sci..

[7] Andabati G, Byamugisha J (2010). Microbial aetiology and sensitivity of asymptomatic bacteriuria among ante-natal mothers in Mulago hospital, Uganda. Afr Health Sci..

[8] Moyo S, Aboud S, Kasubi M, Maselle SY (2010). Bacteria isolated from bloodstream infections at a tertiary hospital in Dar es Salaam, Tanzania—antimicrobial resistance of isolates. S Afr Med J.

[9] Mbanga J, Dube S, Munyanduki H (2010). Prevalence and drug resistance in bacteria of the urinary tract infections in Bulawayo province, Zimbabwe. East Afr J Public Health..

[10] Cheesbrough M (2006). District laboratory practice in tropical countries.

[11] Bauer A, Kirby W, Sherris J, Turck M (1966). Antibiotic susceptibility testing by a standardized single disk method. Am J Clin Pathol.

[12] Institute Clinical Laboratory Standard (2014). Twentey-fourth informational supplement.

[13] Obeng-nkrumah N, Twum-danso K, Krogfelt KA, Newman MJ (2013). High levels of extended-spectrum beta-lactamases in a major teaching hospital in Ghana: the need for regular monitoring and evaluation of antibiotic resistance. Am J Trop Med Hyg.

[14] Leski TA, Taitt CR, Bangura U, Stockelman MG, Ansumana R, Cooper WH, Stenger DA, Vora GJ (2016). High prevalence of multidrug resistant Enterobacteriaceae isolated from outpatient urine samples but not the hospital environment in Bo, Sierra Leone. BMC Infect Dis..

[15] Demilie T, Beyene G, Melaku S, Tsegaye W (2012). Urinary bacterial profile and antibiotic susceptibility pattern among pregnant women in North West Ethiopia. Ethiop J Health Sci.

[16] Nkrumah NO, Labi AK, Addison NO, Ewuramma J, Labi M, Mensah GA (2016). Trends in paediatric and adult bloodstream infections at a Ghanaian referral hospital: a retrospective study. Ann Clin Microbiol Antimicrob..

[17] Mulu W, Abera B, Yimer M, Hailu T, Ayele H, Abate D (2017). Bacterial agents and antibiotic resistance profiles of infections from different sites that occurred among patients at Debre Markos Referral Hospital, Ethiopia: a cross sectional study. BMC Res Notes..

[18] Hackman HK, Brown CA, Twum-danso K (2014). Antibiotic resistance profile of non-extended spectrum beta-lactamase-producing *Escherichia coli* and *Klebsiella pneumoniae* in Accra, Ghana. J Biol Agric Health..

[19] Lerbech AM, Opintan JA, Bekoe SO, Ahiabu MA, Tersbøl BP, Hansen M, Brightson KT, Ametepeh S, Frimodt-Møller N, Styrishave B (2014). Antibiotic exposure in a low-income country: screening urine samples for presence of antibiotics and antibiotic resistance in coagulase negative staphylococcal contaminants. PLoS ONE.

[20] Ntirenganya C, Manzi O, Muvunyi CM, Ogbuagu O (2015). High prevalence of antimicrobial resistance among common bacterial isolates in a tertiary healthcare facility in Rwanda. Am J Trop Med Hyg.

