# Supplementary material for: Antimicrobial resistance among clinically relevant bacterial isolates in Accra: a retrospective study
Source: BMC Res Notes. 2018 Apr 25;11:254. doi: 10.1186/s13104-018-3377-7 (PMC5918760; doi:10.1186/s13104-018-3377-7)
Supplement: Supplementary file 1 — Additional file 1. Antibiotic resistant bacteria isolated from different sample types at the six laboratories. Total number and percentage of resistant bacterial isolates from each type of specimen isolated at TH (Trust Hospital); MDC (Mediplast Diagnostic Center); PSGL (Patholab Solutions Ghana Limited); LAGH (LA General Hospital); G2ML (G2 Medical Laboratory); HTMC (Holy Trinity Medical Center). [file 13104_2018_3377_MOESM1_ESM.docx]

**Additional File 1: Antibiotic resistant bacteria isolated from different sample types at the six laboratories.**

| **Laboratories** | **Specimens** | **Resistant isolates (%)** |
| --- | --- | --- |
| **TH (N=204)** | Wound swab | 33 (16.2^b^) |
|  | Urine | 141(69.1^a^) |
|  | Vaginal swab | 9 (4.4^c^) |
|  | Ear swab | 7 (3.4^cd^) |
|  | Blood | 6 (2.9^cd^) |
|  | Urethral smear | 3 (1.5^cd^) |
|  | Pus fluid | 1 (0.5^de^) |
|  | Sputum | 4 (2.0^cd^) |
| **MDC (N=36)** | Blood | 2 (5.6^b^) |
|  | Wound swab | 4 (11.1^b^) |
|  | Vaginal swab | 6 (16.7^b^) |
|  | Throat swab | 1 (2.8^b^) |
|  | Urine | 19 (52.8^a^) |
|  | Seminal fluid | 2 (5.6^b^) |
|  | Urethral swab fluid | 2 (5.6^b^) |
| **PSGL (N=350)** | Urine | 183 (52.3^a^) |
|  | Vaginal swab | 63 (18.0^b^) |
|  | Blood | 15 (4.3^d^) |
|  | Urethral swab | 38 (10.9^c^) |
|  | Ear swab | 18 (5.1^d^) |
|  | Palate | 1 (0.3^f^) |
|  | Wound swab | 16 (4.6^d^) |
|  | Semen | 10 (2.9^de^) |
|  | Aspirate | 1 (0.3^f^) |
|  | Sputum | 3 (0.9^ef^) |
|  | Pus | 1 (0.3^f^) |
|  | Anal swab | 1 (0.3^f^) |
| **LAGH (N=298)** | Sputum | 9 (3.0^def^) |
|  | Vaginal swab | 27 (9.1^c^) |
|  | Wound swab | 58 (19.5^b^) |
|  | Ear swab | 34 (11.4^c^) |
|  | Urethral swab | 7 (2.3^efg^) |
|  | Pus | 3 (1.0^efg^) |
|  | Eye swab | 7 (2.3^efg^) |
|  | Surgical swab | 6 (2.0^efg^) |
|  | Cord swab | 10 (3.4^de^) |
|  | Exudate | 2 (0.7^fg^) |
|  | Aspirate | 1 (0.3^gh^) |
|  | Abscess | 1 (0.3^gh^) |
|  | Blood | 20 (6.7^cd^) |
|  | Urine | 113 (37.9^a^) |
| **G2ML (N=219)** | Sputum | 4 (1.8^e^) |
|  | Wound | 56 (25.6^b^) |
|  | Urethral swab | 13 (5.9^cd^) |
|  | Urine | 116 (53.0^a^) |
|  | Aspirate | 1 (0.5^e^) |
|  | Ear swab | 16 (7.3^c^) |
|  | Blood | 5 (2.3^de^) |
|  | Cord swab | 1 (0.5^e^) |
|  | Semen | 2 (0.9^e^) |
|  | Vaginal swab | 1 (0.5^e^) |
|  | Eye swab | 2 (0.9^e^) |
|  | Discharge | 1 (0.5^e^) |
|  | Throat swab | 1 (0.5^e^) |
| **HTMC (N=594)** | Urine | 369 (62.1^a^) |
|  | Wound | 7 (1.2^ef^) |
|  | Semen | 10 (1.7^e^) |
|  | Sputum | 117 (19.7^b^) |
|  | Vaginal swab | 50 (8.4^c^) |
|  | Urethral swab | 26 (4.4^d^) |
|  | Ear swab | 5 (0.8^ef^) |
|  | Throat swab | 8 (1.3^ef^) |
|  | Eye swab | 2 (0.3^f^) |

TH: Trust Hospital, MDC: Mediplast Diagnostic Center, PSGL: Patholab Solutions (Ghana) Limited, LAGH: LA General Hospital, G2ML: G2 Medical Laboratory Service, HTMC: Holy Trinity Medical Centre. For each lab, Proportions followed by different letters in a column means difference in proportion of resistant isolates at α<0.05%
